# Supplementary material for: Trends and predictors of change of unmet need for family planning among reproductive age women in Ethiopia, based on Ethiopian demographic and health surveys from 2005–2016: Multivariable decomposition analysis
Source: PLoS One. 2022 Aug 18;17(8):e0272701. doi: 10.1371/journal.pone.0272701 (PMC9387833; doi:10.1371/journal.pone.0272701)
Supplement: Attachment — Submitted filename: PLOS comments.docx [file pone.0272701.s001.docx]

This was an interesting paper, addressing an important topic using generally appropriate methods and a sound data set. However, there were a number of issues with the paper, including formulation of the models, discussion of the results, and quality of the prose and editing which mean that it would need a considerable amount of work for it to be suitable for publication.

1. One of my main areas of concern revolves around the definition of the dependent variable, lines 97-102;

“The outcome variable was unmet need for FP, where it composed of unmet need for spacing and limiting. It refers to the proportion of women who desire to either delay the next pregnancy or limit future pregnancies but are not using any method of modern method of contraception (11). The outcome variable was categorized as “unmet need” if women had unmet need for spacing and limiting were and coded as 1, while those using FP methods for spacing or limiting or with no unmet need were “met need” coded as 0”.

But to my understanding, unmet need considers not only sexually active women who desire to delay/limit future pregnancies but it includes those who were currently pregnant and gave birth recently, whether the pregnancy or recent birth was wanted or not (Look the framework below). How do you justify this?

2. My next concern is, Abebaw et al, 2015 tried to address the issue with a similar procedure and dataset see, <https://journals.plos.org/plosone/article/comments?id=10.1371/journal.pone.0116525>

What is your contribution?

3. The discussion would benefit from a clearer distinction between contributions to the unmet need change as found here to result from changes in 1) the characteristics of women and 2) the correlates (or effects) of these characteristics with (on) contraceptive use.

Finally, in my opinion, the manuscript could be considered for publication but prior to publication minor revisions are suggested to improve the manuscript.
