# Supplementary material for: Trends and predictors of change of unmet need for family planning among reproductive age women in Ethiopia, based on Ethiopian demographic and health surveys from 2005–2016: Multivariable decomposition analysis
Source: PLoS One. 2022 Aug 18;17(8):e0272701. doi: 10.1371/journal.pone.0272701 (PMC9387833; doi:10.1371/journal.pone.0272701)
Supplement: Attachment — Submitted filename: response to reviewers.docx [file pone.0272701.s003.docx]

Authors’ response to reviews

Title: Trends and Predictors of Change of Unmet Need for Family Planning among Reproductive Age Women in Ethiopia , based on Ethiopian Demographic and Health Surveys from 2005-2016: multivariable Decomposition Analysis

Authors:

Abiyu Abadi Tareke (abiyu20010@gmail.com)

Ermias Bekele Enyew ([Ermiashi@gmail.com](mailto:Ermiashi@gmail.com))

Berhanu Fikadie Endeabatu ([berhanufikadie@gmail.com](mailto:berhanufikadie@gmail.com))

Abiy Tasew Dubale(abiytasewhi@gmail.com)

Habitu Birhan Eshetu (sisay419@gmail.com)

Sisay Maru Wubante(sisay419@gmail.com)

Version: 1 Date: 10 July, 2022

Author’s response to reviews:

BMC Health Services Research

Point by point response for editors/reviewers comments

Manuscript title: Trends and Predictors of Change of Unmet Need for Family Planning among Reproductive Age Women in Ethiopia , based on Ethiopian Demographic and Health Surveys from 2005-2016: multivariable Decomposition Analysis

Manuscript ID: ccc25ca3-db68-48c6-87d6-65d9d0770161

Dear editor/reviewer:

Dear all,

We would like to give deep gratitude for your constructive and fruitful comments. You valuable comments enhanced content of the manuscript and we got highly experienced of scientific paper writing. The authors accounted each comments and clarification questions of editors and reviewers in a focused way. Our point-by-point responses to each comment and questions are detailed on the following pages. Further, the details of changes were shown under track changes in the supplementary document attached.

Response to Editors and reviewers’ comments

**Editors’ request:** The name of the colleague who edited your manuscript?

**Authors’ response:** The name of the colleague who helped us in editing and improving the English language of our manuscript was Bayley Adane Takele. He has MPH in biostatistics and 5 years’ experience in scientific paper writing specially in health related manuscript writing.
